# Eukaryotic protein production in designed storage organelles

**DOI:** 10.1186/1741-7007-7-5

**Published:** 2009-01-28

**Authors:** Margarita Torrent, Blanca Llompart, Sabine Lasserre-Ramassamy, Immaculada Llop-Tous, Miriam Bastida, Pau Marzabal, Ann Westerholm-Parvinen, Markku Saloheimo, Peter B Heifetz, M Dolors Ludevid

**Affiliations:** 1 Departament de Genètica Molecular, Consorci CSIC-IRTA, Jordi Girona, 08034 Barcelona, Spain; 2 ERA Biotech, S.A. Parc Científic de Barcelona, Baldiri Reixac, 08028 Barcelona, Spain; 3 VTT Technical Research Centre, PO Box 1000, FIN-02044VTT, Finland

## Abstract

**Background:**

Protein bodies (PBs) are natural endoplasmic reticulum (ER) or vacuole plant-derived organelles that stably accumulate large amounts of storage proteins in seeds. The proline-rich N-terminal domain derived from the maize storage protein γ zein (Zera) is sufficient to induce PBs in non-seed tissues of Arabidopsis and tobacco. This Zera property opens up new routes for high-level accumulation of recombinant proteins by fusion of Zera with proteins of interest. In this work we extend the advantageous properties of plant seed PBs to recombinant protein production in useful non-plant eukaryotic hosts including cultured fungal, mammalian and insect cells.

**Results:**

Various Zera fusions with fluorescent and therapeutic proteins accumulate in induced PB-like organelles in all eukaryotic systems tested: tobacco leaves, *Trichoderma reesei*, several mammalian cultured cells and Sf9 insect cells. This accumulation in membranous organelles insulates both recombinant protein and host from undesirable activities of either. Recombinant protein encapsulation in these PBs facilitates stable accumulation of proteins in a protected sub-cellular compartment which results in an enhancement of protein production without affecting the viability and development of stably transformed hosts. The induced PBs also retain the high-density properties of native seed PBs which facilitate the recovery and purification of the recombinant proteins they contain.

**Conclusion:**

The Zera sequence provides an efficient and universal means to produce recombinant proteins by accumulation in ER-derived organelles. The remarkable cross-kingdom conservation of PB formation and their biophysical properties should have broad application in the manufacture of non-secreted recombinant proteins and suggests the existence of universal ER pathways for protein insulation.

## Background

Efficient expression, accumulation and recovery of recombinant eukaryotic proteins in their native conformations are difficult to achieve at reasonable cost in cell-based biomanufacturing systems [1]. In general, such platforms are either highly capital-intensive mammalian cell cultures based on secreted proteins [2], or require complex and inefficient refolding of bacterially expressed proteins sequestered in insoluble inclusion bodies [3]. Among eukaryotes, the plant kingdom has evolved cereal seeds to facilitate massive and extremely stable intracellular accumulation of complex proteins in a dense form that enables rapid protein mobilization during germination. A key biological sequestration mechanism of plant seed storage proteins involves the formation of protein bodies (PBs) [4-6]. In maize, one type of PB formed in the endoplasmic reticulum (ER) lumen of endosperm cells [7] contains zeins, a group of polypeptides which account for more than half of the total seed protein mass. The 27 kD γ zein protein bears an N-terminal signal sequence but is retained intracellularly rather than being secreted even though it lacks a canonical KDEL/HDEL ER-retention signal. Instead, γ zein localizes to the periphery of the PBs surrounding aggregates of other zein proteins [8,9]. Although the mechanism of PB biogenesis is not fully understood, it appears that the initial presence of γ zein confers stability to other PB-associated zeins and facilitates their sequential assembly in the PB [10]. Surprisingly, heterologous expression of γ zein in plant leaves results in the ectopic formation of membrane-bound structures strongly resembling cereal PBs [11,12], suggesting that one or more structural motifs of γ zein may be responsible for *de novo *PB formation. This hypothesis is supported by the finding that a synthetic peptide of the N-terminal repeat region of γ zein (eight repeating units of the peptide PPPVHL) was able to self-assemble *in vitro *[13] and that both γ zein proline-rich domains, the repeat region and the so-called Pro-X sequence, each contribute to PB biogenesis when expressed recombinantly in plants [12,14].

Here, we describe a protein fusion-based approach for heterologous protein production via creation of induced PB-like organelles. Exploiting the natural mechanism of protein accumulation in seed PBs, we demonstrate that not only is the Zera domain capable of directing assembly of PB-like organelles in plant tissues other than seeds, but this assembly property also extends to a broad range of non-plant eukaryotes including fungal, insect and mammalian cells. These synthetic PBs share many of the advantageous storage properties of bona fide cereal PBs, including the presence of eukaryotic chaperones to facilitate recombinant protein folding, high densities that allow simple downstream target protein concentration by PB capture, and insulation of PB contents from proteolytic and enzymatic activities of the cytosol. Transferring the seed-type attributes conferred by PB sequestration to proven eukaryotic cell-based and transgenic expression systems creates unique opportunities for improving biological manufacturing. Such an approach is of particular interest for polypeptides which are poorly secreted, host-toxic, or otherwise challenging to produce.

## Results

### The γ zein proline-rich domain (Zera) induces protein body-like organelles in eukaryotic cells

**Figure 1:**
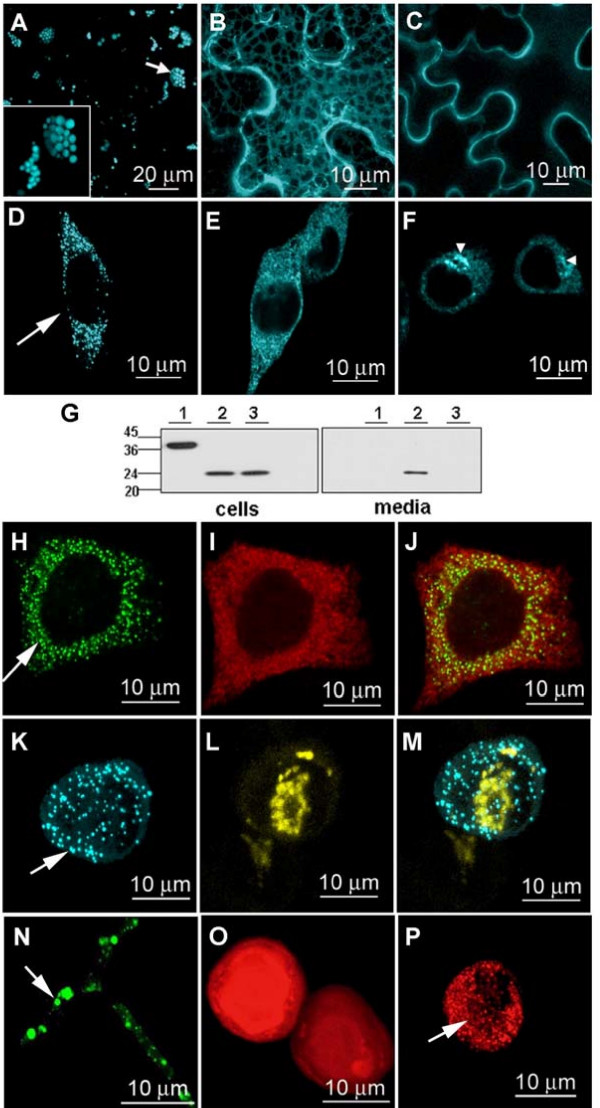
**Proline-rich domain of γ zein (Zera) induces PB-like organelles in plants, CHO cells, insect cells and fungi**. **(A) **Confocal image of PB-like organelles formed in epidermal leaf cells of tobacco transformed with Zera-ECFP (see a higher magnification image in the inset). **(B) **ER network image of ECFP retained in the ER of tobacco cells expressing SPg-ECFP-KDEL. **(C) **ECFP secretion pattern in tobacco cells transformed with SPg-ECFP construct. (**D**) PB-like organelles (arrow) in CHO cells transfected with Zera-ECFP construct. (**E**) ER pattern in SPgECFP-KDEL expressing CHO cells. **(F) **Fluorescent ER and Golgi complex (arrowheads) in SPgECFP expressing CHO cells denoting ECFP secretion. **(G) **Immunoblot using an anti-GFP antibody of cell extracts and media of CHO cultured cells expressing Zera-ECFP (lanes 1), ECFP preceded by the γ zein N-terminal signal peptide (SPg-ECFP) (lanes 2) and ECFP-KDEL (from SPg-ECFP-KDEL construct) (lanes 3). CHO cells expressing both, Zera-GFP **(H) **and calnexin-DsRed (ER membrane marker **(I) **show co-localization of both proteins (merge in **J**). Co-expression of Zera-ECFP **(K) **and a glycosyltranferase labelled with YFP (Golgi reporter in yellow, **L**), does not result in these proteins co-localization (merge in **M**). **(N) **Hyphal filaments of *Trichoderma reesei *transformed with Zera-EGFP showing green fluorescent PB-like organelles within hyphae (arrow). (**O, P) **Baculovirus-mediated expression of DsRed **(O) **and Zera-DsRed **(P) **in Sf9 cells showing the induction of red fluorescent PBs (arrow in **P**).

To explore if PB attributes could be transferred to divergent eukaryotic lineages and a broad range of fusion protein partners, we first investigated the induction of PBs in various hosts expressing fusions of the engineered Zera domain to fluorescent reporter proteins. Epidermal cells of tobacco leaves transiently expressing a Zera-ECFP (enhanced cyan fluorescent protein) construct displayed well-defined and highly fluorescent foci distributed throughout the cells (Figure 1A) that were similar in size and appearance (Figure 1A, see inset) to the natural, round, 1 to 2 micron PBs present in maize seeds [9]. To define more specifically the role of Zera in PB formation we compared these results with the fluorescence of ECFP preceded by the γ zein N-terminal signal peptide either with (SPg-ECFP-KDEL, Figure 1B) or without (SPg-ECFP, Figure 1C) the KDEL C-terminal ER retention signal. The proteins from construct SPg-ECFP-KDEL accumulated in the ER (Figure 1B) while those from SPg-ECFP were secreted (Figure 1C), but neither were capable of inducing PB-like organelles.

We next investigated the properties of Zera fusions expressed in animal cells. Chinese hamster ovary (CHO) cells were transfected stably and transiently with Zera-ECFP constructs or appropriate non-Zera controls. Dense, highly fluorescent foci became visible in the cytoplasm starting two to three days following transfection with Zera-ECFP (Figure 1D). The contribution of the Zera domain to storage organelle formation was also investigated using ECFP fused directly to SPg, either with or without a C-terminal KDEL retention signal. Confocal images obtained from cells expressing SPg-ECFP-KDEL showed a typical ER pattern (Figure 1E), while fluorescence was distributed throughout the ER and the Golgi complex (Figure 1F, arrow heads) as expected for secretory transport in cells expressing SPg-ECFP without KDEL. We also analyzed by immunoblotting the effect of Zera on partitioning of ECFP between cells and the extracellular medium (Figure 1G). Zera-ECFP and ECFP-KDEL remained exclusively intracellular (Figure 1G, lanes 1 and 3), while ECFP without Zera or KDEL was secreted efficiently (Figure 1G, lanes 2). Taken together these results show evidence for the entry of Zera-ECFP into the initial portion of the secretory pathway but exclusion from ultimate secretion. This observation prompted us to investigate whether Zera-ECFP was sorted terminally in the ER, or was able to exit this compartment in order to subsequently reach the Golgi complex. Zera fusions were co-transfected with an ER membrane protein marker, calnexin, and with a Golgi protein reporter, a glycosyltransferase fused to YFP. Calnexin was found to co-localize with Zera-ECFP PBs (Figure 1H to 1J) but no co-localization was observed in CHO cells between Zera-ECFP and the Golgi reporter protein (Figure 1K to 1M), consistent with an ER origin of the Zera-induced PBs.

Filamentous fungi and insect cells were also found to support the biogenesis of seed-type PBs induced by expression of Zera fusions. Significant accumulation of Zera-GFP fusion protein in dense structures was observed in hyphae of *Trichoderma reesei *transformed with Zera-GFP constructs (Figure 1N, arrow). The Zera fusion protein accumulated intracellularly to ca. 0.1% of fresh weight when expression was driven by the Trichoderma *CbhI *promoter. In Spodoptera (Sf9) cells infected with recombinant Zera-DsRED baculovirus, the fusion protein also accumulated in abundant, intensely-fluorescent PB-like structures (Figure 1P, arrow). In contrast, DsRed controls expressed without Zera showed fluorescence dispersed diffusely throughout the cells with no formation of dense structures (Figure 1O).

### Zera stabilizes biopharmaceutical proteins by intracellular encapsulation in PB-like organelles

**Figure 2:**
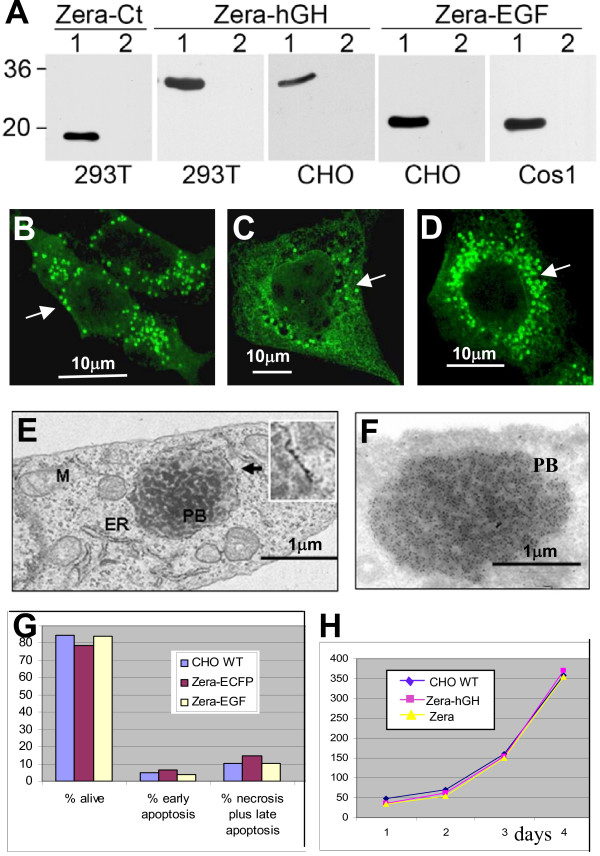
**Several biopharmaceutical proteins fused to Zera remain encapsulated in PB-like organelles in transfected mammalian cells**. **(A) **Western blots of Zera-Ct, Zera-hGH and Zera-EGF demonstrating accumulation inside transfected mammalian cells (lanes 1) without significant secretion into the cell culture media (lanes 2). **(B-D) **Immunodetection of Zera-Ct in 293T **(B)**, Cos1 **(C) **and CHO **(D) **transfected cells using an anti-calcitonin antibody and FITC-labeled secondary antibody. Green fluorescence indicates recombinant protein accumulation in PB-like organelles in all three cases. **(E, F) **Electron microscopy images of a CHO cell line stably transfected with Zera-hGH; **(E) **CHO cell showing a PB-like organelle (PB) containing electron-dense structures surrounded by a classical rough-ER membrane (see ribosomes in inset). **(F) **Immunodetection of Zera-hGH recombinant protein in PBs induced in transfected CHO cells. Cryosections were incubated with an anti-hGH antibody and labeled with Protein A-gold particles (10 nm). PB, protein body-like organelle; M, mitochondria; ER, endoplasmic reticulum. **(G) **Viability of CHO cells stably transfected with Zera-ECFP and Zera-EGF. Relative percentages of living, necrotic and apoptotic transfected cells were compared to those of non-transfected controls (CHO WT). **(H) **Proliferation profiles of CHO cells expressing Zera-hGH and Zera polypeptides as compared to non-transfected CHO WT cells over 1 to 4 days of incubation.

The ability of the Zera domain to segregate proteins away from normal secretory transport into PB organelles appears to be conserved between plants, fungi, insects and animals. In light of these results, we then asked whether Zera fusions could provide an efficient and universal means to produce diverse recombinant proteins by stable encapsulation in ER-derived organelles. To test this hypothesis, we fused Zera to calcitonin (Ct), epidermal growth factor (EGF) and human growth hormone (hGH) sequences and expressed these fusion constructs in various eukaryotic hosts. Expression and PB induction by Zera-Ct, Zera-EGF and Zera-hGH was first investigated using three different types of mammalian cells following transient or stable transfection (Figure 2A). Human HEK293T cells were transfected with Zera-Ct and Zera-hGH, CHO cells with Zera-hGH and Zera-EGF and Cos1 cells with Zera-EGF. In all cases, Zera fusion proteins were present intracellularly (Figure 2A, lanes 1) but were not secreted (Figure 2A, lanes 2). Moreover, immunodetection of calcitonin in transiently transfected human (HEK293T), primate (Cos1) and hamster (CHO) cells expressing Zera-Ct revealed that the recombinant protein was encapsulated inside PB-like vesicles in all cultured cell types tested (Figure 2B to 2D).

We also generated CHO cell lines stably expressing Zera-hGH. The recombinant protein had the expected molecular weight and was localized to prominent electron-dense spherical structures that were 1 to 2 microns in size (Figure 2E to 2F). The membrane bounding the PB-like structures in Zera-hGH CHO cells was studded with ribosomes (Figure 2E inset), suggestive of a rough-ER origin, and the structures were highly enriched in Zera-hGH (Figure 2F). Immunogold-labeled Zera-hGH appeared in the newly formed PBs, whereas no significant labeling was observed in any other cell compartment. Cryosections of non-transfected cells did not contain PB-like structures (data not shown). Interestingly, despite the presence of heterologous PB organelles containing high concentrations of recombinant proteins, transfected mammalian cells were indistinguishable from non-transfected CHO cells with respect to proliferation ability and propensity to undergo apoptosis or necrosis (Figure 2H and 2G, respectively). This indicates that the accumulation of Zera fusion proteins within PBs does not in itself perturb normal cell growth and viability.

**Figure 3:**
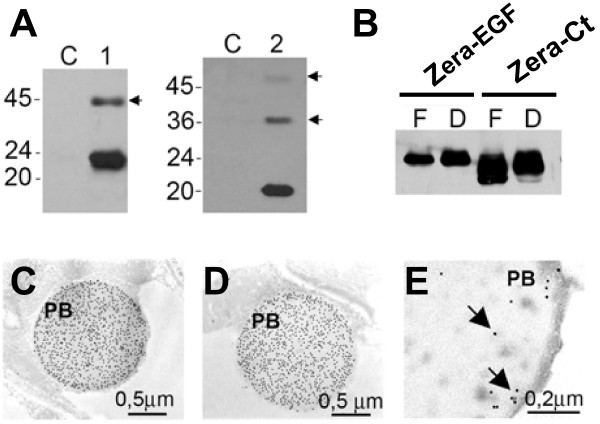
**Zera stabilizes biopharmaceutical proteins in transformed plants**. **(A) **Immunodetection with anti-Zera antibody (anti-R8) of Zera-EGF (lane 1) and Zera-Ct (lane 2) in leaves of stably-transformed tobacco plants. Arrows indicate oligomers, and C denotes lanes containing proteins from non-transformed controls. **(B) **Stability of Zera-EGF and Zera-Ct fusion proteins in desiccated leaves of transgenic tobacco plants. Protein extracts from equivalent amounts of tissue were loaded in each lane and relative accumulation was visualized by anti-Zera immunoblotting of protein extracts from fresh (F) and desiccated (D) leaves. **(C-E) **Immunogold labeling of Zera-Ct inside newly formed PBs in transformed tobacco leaves using anti-R8 **(C)**, anti-calcitonin **(D) **and anti-BiP **(E**) antibodies. PB, induced PBs.

To explore the ability of induced PBs to accumulate biopharmaceutical recombinant proteins in plants we generated transgenic tobacco expressing Zera-EGF and Zera-Ct fusions. Protein extracts from leaves of homozygous Zera-EGF and Zera-Ct tobacco plants were analyzed by immunoblotting using an antibody (αR8) raised against Zera. Major bands corresponding to the expected apparent molecular masses of the respective fusion proteins were observed (Figure 3A, lanes 1 and 2), with fainter high MW bands (Figure 3A, arrows) likely reflecting oligomerized Zera fusions. Similar oligomers have been observed for native maize γ zein and γ zein proline-rich domains when expressed in *Arabidopsis *[12] as well as in protein extracts from alfalfa plants and in NT-1 tobacco cells expressing Zera fusions to the F1-V antigen of *Yersinia pestis *(G Cardineau, Arizona State University, personal communication). In order to evaluate the stability of Zera-EGF and Zera-Ct fusions in PBs, we compared protein accumulation in flash-frozen leaf material vs. leaves dried at 37°C for one week after harvest and subsequently stored at room temperature for five months (such storage typically results in substantial proteolysis). At the end of the five-month storage period expression levels of each fusion protein were determined by immunoblotting. No significant difference was found between frozen and dried leaves (Figure 3B), despite the significant desiccation stress.

Zera-Ct was localized in leaf mesophyl cells of transgenic plants (Figure 3C to 3E) within 1 to 2-micron membrane-bound organelles that were heavily decorated with immunogold particles targeting either the Zera domain (Figure 3C) or Ct (Figure 3D). No significant immunolabeling was observed in the cell wall, nucleus, chloroplasts or other organelles. The major ER chaperone BiP was also detectable by immunogold within the PB-like structures (Figure 3E) suggesting that the process of PB formation may involve incorporation of pre-existing ER-resident proteins into these structures. BiP is known to be present in cereal PBs where it is thought to play a role in storage, protein folding and assembly [15]. Hence, co-localization of Zera-Ct and BiP in the PB-like organelles formed in tobacco leaves indicates that the recombinant fusion proteins are associated closely with chaperones that may mediate eukaryotic protein folding in the ER. The transgenic plants grew and developed normally and were not adversely affected despite the presence of PB organelles and large amounts of fusion protein.

**Figure 4:**
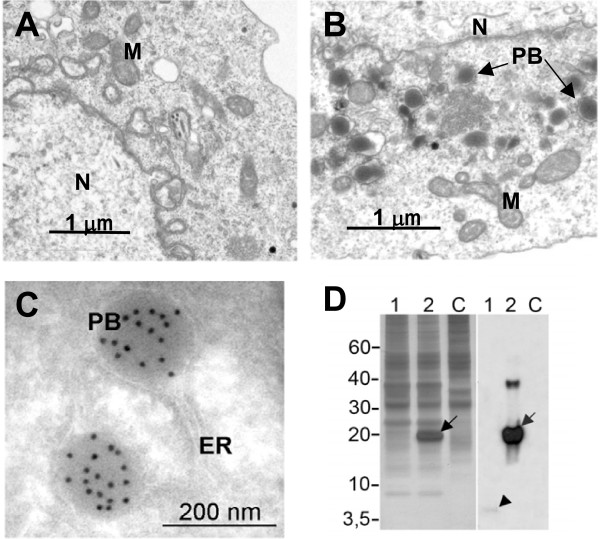
**Sf9 insect cells accumulate high levels of Zera fusions in induced PBs**. **(A-C) **Electron micrographs of control and Zera-EGF Sf9 cells. **(A) **Cells immediately post-infection. **(B) **Electron-dense PB-like structures (PB) visible at 2 days post-infection. **(C) **Immunogold labeling of Zera-EGF expressing Sf9 cells using anti-EGF antisera. N: nuclei; M: mitochondria; ER. endoplasmic reticulum; PB: PB-like organelles. **(D) **Silver stained SDS-PAGE (left) and immunoblot (right) of protein extracts from Sf9 cells expressing Zera-EGF (lanes 2), expressing EGF alone (lanes 1) and non-infected control cells (lanes C). Note the high accumulation of Zera-EGF in lanes 2 (arrows) compared with equivalent non-Zera EGF-expressing cell extracts (lane 1, arrowhead).

We investigated the accumulation of EGF in insect cells by baculovirus infection with Zera-EGF. Sf9 cells accumulated Zera-EGF to remarkably high levels, exceeding 15% of total protein (Figure 4D lane 2). In contrast, EGF accumulated to much lower levels (Figure 4D lane l) in cells infected with virus containing constructs lacking Zera. In fact, EGF in these cells was not detectable by silver or Coomassie staining, and was only faintly visualized by immunoblot (Figure 4D arrowhead). Cryosections of Zera-EGF infected cells (Figure 4B) showed the presence of abundant electron-dense structures which were absent in non-infected Sf9 cells (Figure 4A). These PB-like particles were heavily decorated by anti-EGF immunogold (Figure 4C).

To evaluate the efficiency of recombinant protein production in induced PBs we compared the accumulation level of recombinant EGF and hGH proteins expressed with and without Zera in tobacco plants and insect cells (Table 1). In both expression systems, fusion with Zera resulted in marked increases in target protein accumulation associated with PB induction.

**Table 1:** Accumulation levels of proteins expressed with Zera and without Zera

	**Tobacco**	**Sf 9 Cells**
	g POI/Kg fresh biomass +/- SD	μg POI/10^6 ^cells +/- SD

**Zera-EGF**	0.5 +/- 0.1	5.6 +/- 2.01

**EGF**	0.005 +/- 0.002	0.5 +/- 0.032

**Zera-hGH**	3.2 +/- 1.39	88 +/- 30.3

**hGH**	0.25 +/- 0.09	6.18 +/- 1.13

POI: Product of interest; SD: standard deviation; EGF and hGH recombinant proteins

### Induced PBs facilitate recombinant protein recovery

**Figure 5:**
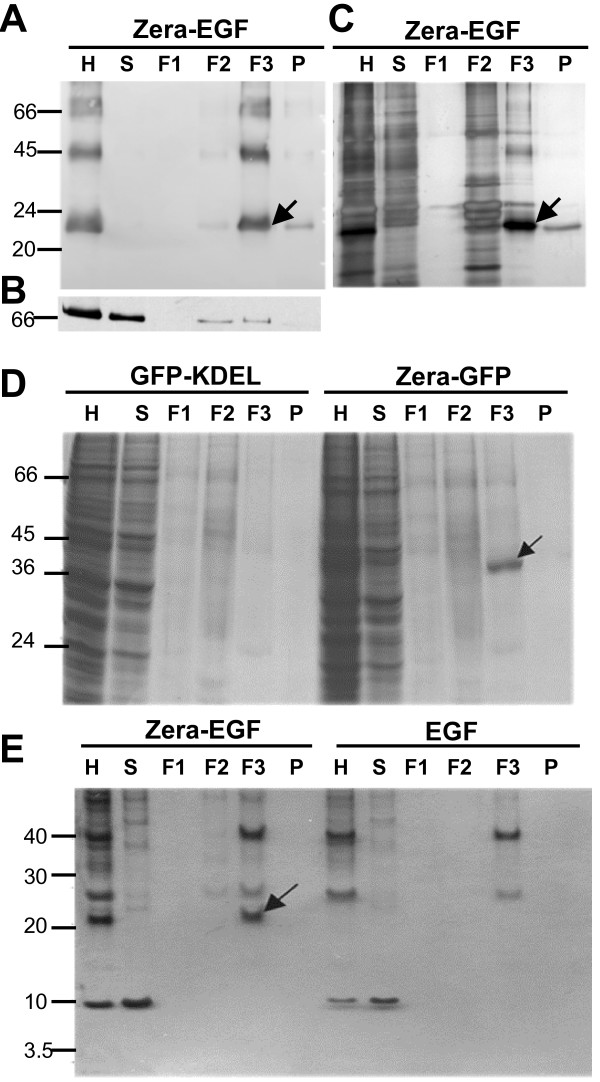
**PBs isolation by density and Zera-fusion proteins recovery**. **(A-C) **Protein analysis of fractions collected from a sucrose gradient of Zera-EGF tobacco leaf homogenates. **(A) **Immunoblot (αR8 antibody) shows Zera-EGF primarily in the dense F3 fraction (1.18 to 1.26 g/cm^3 ^interface, arrow). **(B) **Immunoblot using anti-BiP antibody indicates that the soluble ER chaperone co-sediments with Zera-EGF in the F3 fraction. **(C) **Silver stained SDS-PAGE of sucrose fractions showing the enrichment of Zera-EGF in the F3 fraction. (**D**) Subcellular fractionation of homogenates of *T. reesei *expressing Zera-GFP (right) and GFP-KDEL (left). The analysis of fractions by Coomassie staining shows highly concentrated Zera-GFP in the dense fraction F3 (1.18 to 1.26 g/cm^3 ^interface, arrow). **(E) **Protein analysis by gel electrophoresis and silver stain of fractions collected from sucrose gradients of Zera-EGF (left) and EGF (right) Sf9 expressing cells. Recombinant Zera-EGF fusion is concentrated in the dense fraction F3 (arrow) where PBs sediment.

One of the most significant bottlenecks in non-secreted recombinant protein production is the recovery of the protein from the expression host. We explored whether Zera-protein fusions could be isolated from crude cell or tissue extracts by virtue of the high density of Zera-induced PBs (Figure 5). Leaf homogenate extracts from Zera-EGF transgenic tobacco plants were fractionated on density gradients and analyzed by gel electrophoresis and immunoblotting using anti-Zera and anti-BiP antibodies (Figure 5A to 5C). Most of the Zera-EGF protein was recovered in the dense 1.18 to 1.26 g/cm^3 ^sucrose gradient interface (Figure 5A and 5C, F3), with some present in the ER fraction (Figure 5A, F2). BiP also partitioned with the PB-containing fraction (Figure 5B, F3), in agreement with the immunoelectron microscopy findings for Zera-Ct (Figure 3E). Zera-protein fusions could also be recovered by density from transformed filamentous fungi and insect cells extracts. In *Trichoderma *(Figure 5D), Zera-GFP was detectable by both direct Coomassie staining and immunoblotting following gel electrophoresis of dense particles isolated on sucrose density gradients. In controls lacking Zera no GFP could be isolated in high-density fractions (Figure 5D, compare lanes F3). As in plants and fungi, dense PB-like structures from Zera-EGF-expressing Sf9 cells were readily isolated by density gradients (Figure 5E). Zera-EGF fusion protein but not EGF could be recovered from crude lysates in the 1.18 to 1.26 g/cm^3 ^sucrose gradient interface (Figure 5E, lanes F3). It should be noted that low-speed direct centrifugation of the homogenates, while less efficient than gradients (which enriched the fusion protein up to 85% in a single density separation following cell lysis) was also able to effect substantial concentration and purification of Zera fusions (data not shown).

## Discussion

Our results indicate that in a variety of eukaryotic cells the N-terminal proline-rich portion of γ zein, the Zera assembler domain, is by itself sufficient to direct recombinant fusion protein accumulation within *de novo*-produced storage organelles similar to the PBs of seeds. These organelles retain many of the beneficial characteristics of cereal seed PBs, including the physical properties of high local concentration within a discrete membrane-bound structure that allows efficient isolation by simple density-based protocols, sub-cellular sequestration that facilitates protein accumulation preventing adverse metabolic interactions, and association with molecular chaperones that may facilitate folding and enable functional and stable expression of enzymes and human proteins. Remarkably, the mechanism by which the ER of cereal seeds segregates storage proteins away from normal secretory transport and accumulates them into PB organelles appears to be conserved between plants, fungi, insects and animal cells. This finding suggests that PB biogenesis may be related to general cis-mediated biophysical interactions of the ER with the Zera structure rather than depending on trans-factor associated processes such as receptor-mediated signaling. The mechanism by which Zera forms these novel cell organelles is not yet fully characterized, but an important contributing factor is likely to be the physico-chemical properties of the PPPVHL repeat domain of Zera. This domain folds *in vitro *into an amphipathic poly-Proline II conformation [13] that in turn forms an expanded helix able to assemble both with itself [16] and with membrane lipids [17]. Besides the specific properties derived from its amphipathic nature, the Zera domain also contains six cysteine residues, which may serve to stabilize nascent assembled Zera complexes via intermolecular disulfide bonds. Studies of PB formation in tobacco plants expressing a chimeric storage protein created by fusion of the maize γ zein proline-rich domain and the legume storage protein phaseolin [14] suggests that γ zein Cys residues contribute to fusion protein polymerization and accumulation in the ER [18].

Recombinant protein accumulation was clearly enhanced by using Zera. This enhancement has been observed with several proteins expressed in different eukaryotic hosts with and without fusion to Zera. Recombinant hGH accumulated 13 times more when transiently expressed as Zera-hGH in *Nicotiana benthamiana*, while the differential accumulation of recombinant EGF as a fusion was 100-fold greater than EGF alone (Table 1). These high levels of fusion protein accumulation in PBs suggest that in addition to organellar sequestration, Zera protein condensation may also provide a mechanism for such fusions to evade the normal ER degradative pathways. The ER-associated degradation (ERAD) mechanism [19,20] is induced transcriptionally in all eukaryotic cells in response to the presence of unfolded proteins [21]. This mechanism is conserved from yeast to humans and affects both endogenous and heterologous proteins. High accumulation of Zera fusions assembled into heterologous PBs may therefore reflect poor recognition by the ERAD pathway and/or inefficient delivery of fusions to the cytosol for proteolysis following ERAD pathway entry.

In addition, accumulation of recombinant Zera fusion proteins apparently does not alter normal host cell growth and development, and neither predisposes such cells to apoptosis nor perturbs the normal functionality of the secretory pathway. It is well known that if the rate of synthesis of a given protein exceeds the rates of folding and degradation the resultant over-accumulation in the ER usually triggers apoptosis [22]. Our results suggest that this is not the case for PB-accumulated proteins, as we observed that 1) transgenic Zera plants germinated, were fertile and developed identically to wild-type plants; 2) apoptosis profiles and proliferation rates of CHO cells over-expressing Zera fusion proteins were similar to non-transfected CHO cells; and 3) fungal cells expressing Zera fusions grew normally and vigorously despite accumulating large intracellular PBs. Naturally occurring ER-derived structures sequestering aberrant proteins have been observed previously in mammals. Such structures include the so-called Russell bodies found in non-apoptotic plasma cells of multiple myeloma and AIDS patients [23]. These structures are thought to arise from dilated ER cisternae that sequester condensed immunoglobulins that might otherwise trigger deleterious cellular responses [24]. The broad species conservation of PB induction may therefore reflect an ancestral protective response to mitigate the potentially adverse effects of protein over-accumulation in the ER. Taken together, our observations of high intracellular accumulation, enhanced recombinant protein stability, and the lack of adverse metabolic consequences for the expressing host cell all suggest that sequestration within membrane-bound PBs serves as an effective means to isolate the cell and recombinant target protein from one another.

An additional key consideration for recombinant protein production is the efficiency of downstream recovery and purification [25]. Packaging of recombinant protein into dense organelles that are easily concentrated from cell lysates provides a significant enrichment of pre-purification titer prior to chromatographic separation or capture steps. The resulting density-fractionated Zera protein fusions can be readily purified following disaggregation under mild reducing conditions. This creates an opportunity to design improved downstream purification protocols that may be less costly than current approaches. Currently, downstream purification comprises the bulk of the costs associated with recombinant protein biopharmaceutical manufacture.

## Conclusion

Zera sequence provides an efficient and universal means to produce recombinant proteins by accumulation in ER-derived organelles. This intracellular storage and sequestration system evades the normal cellular protein degradation mechanisms and does not perturb cell metabolism. The PB structures formed by Zera fusions share many of the advantageous storage properties of bona fide cereal seed PBs, including the presence of eukaryotic chaperones to facilitate recombinant protein folding, high densities that allow simple downstream target protein concentration by PB capture, and insulation of PB contents from proteolytic and enzymatic activities of the cytosol. Transferring the seed-type attributes conferred by PB sequestration to proven eukaryotic cell-based and transgenic expression systems creates unique opportunities for improving biological manufacturing. Such an approach is of particular interest for polypeptides which are poorly secreted, host-toxic, or otherwise challenging to produce.

## Methods

### Plasmid constructs

Plasmid pUC18Zera coding for the Zera N-terminal γ zein coding sequence (Zera, RX3 sequence) [26] was obtained after amplification of the DNA sequence coding for the Zera domain by PCR using the vector pKSG2 [27] as template and suitable oligonucleotides as primers. The synthetic genes and cDNA sequences for mature calcitonin (Ct), epidermal growth factor (EGF), human growth hormone (hGH) and cyan (ECFP), green (EGFP) and red (DsRED) fluorescent proteins were fused to the 3' end of Zera N-terminal γ zein-coding sequence in the plasmid pUC18Zera. When desired, sequences coding for specific protease cleavage sites (Factor Xa or Enterokinase) were included between Zera and fused gene sequences.

To transform mammalian cells the fusion protein coding sequences Zera-Ct, Zera-EGF, Zera-hGH and Zera-ECFP from pUC18-derived plasmids were introduced in the mammalian transfection vector pcDNA3.1(-) (Invitrogen) under the human cytomegalovirus immediate-early (CMV) promoter to give the constructs p3.1ZeraCt, p3.1ZeraEGF, p3.1ZerahGH and p3.1ZeraECFP. Plasmid p3.1ZeraSTOP was used to express the Zera polypeptide alone and contained the Zera cDNA with a stop codon at the 3' end. Finally, plasmid pECFP-N1 (Clontech) was used as the template to obtain SPg-ECFP and SPg-ECFPKDEL protein coding sequences by fusion of the γ zein signal peptide (SPg) with ECFP sequences containing or not containing the KDEL sequence at the 3' end, using two 5' overlapping primers and suitable reverse primers. PCR products were introduced in pCR-Blunt (Invitrogen) and the new plasmids were named pCRSPgECFP and pCRSPgECFPKDEL. Finally the mammalian transfection vectors p3.1SPgECFP and p3.1SPgECFPKDEL were obtained.

For plant transformation, the Zera-ECFP, SPg-ECFP and SPg-ECFPKDEL protein-coding sequences were introduced into the binary plant vector pCambia 2300  and the Zera-Ct and Zera-EGF-coding DNAs were introduced into the binary vector pBin19. All fusion sequences were under the control of the enhanced 35S cauliflower mosaic virus (CaMV) promoter and the resulting plant transformation vectors were named, respectively, pCZeraECFP, pCSPgECFP, pCSPgECFPKDEL, p19ZeraCt and p19ZeraEGF.

For insect cells infection, Zera-EGF and Zera-DsRED DNA sequences were introduced into the vector pBacPak8 (Clontech) to obtain vectors pBacPak8ZeraEGF and pBacPak8ZeraDsRED. Plasmids pBacPak8EGF and pBacPak8DsRED were used as controls for EGF and DsRED non-fused protein expression in insect cells. The construct containing the coding sequence of an improved monomeric Ds Red protein (mCherry) was kindly provided by Dr RY Tsien [28].

Two constructs for expression of EGFP in *Trichoderma reesei *were created in the plasmid pMS186 essentially as described [29] using the plasmids pUC18Zera and pSM1-EGFP [30] as templates in PCR. The forward primer encoded the *cbhI *signal sequence that was fused to the 5' terminus of the Zera peptide sequence in the new pMS186ZeraGFP construct containing the Zera-EGFP coding sequence, whereas the EGFP control construct pMS186GFPHDEL included the HDEL C-terminal ER retention signal sequence.

### Cell culture, transfections and transformations of mammalian cell culture, fungi, plants and insect cells

#### Mammalian cell cultures

Cos 1 and 293T cells were grown in DMEM medium (GIBCO) and CHO cells in HAM F-12 (GIBCO) medium, both media supplemented with 10% fetal calf serum (PAA) and 2 mM L-Glutamine (Sigma). Monolayer cultures were maintained at 37°C, 5% CO_2 _and 100% relative humidity. Stable and transient transfection of cultured cells was done by the lipofectamine-based method (Invitrogen) according to the manufacturer's instructions. Stably transfected CHO clonal cell lines expressing the protein fusions Zera-hGH and Zera-ECFP were selected in the supplemented HAM F-12 medium containing 500 μg/ml geneticin (Invitrogen).

#### Plants

*Nicotiana tabacum *(var. Wisconsin 38) plants were stably transformed with *Agrobacterium tumefaciens *containing the binary vectors p19ZeraCt or p19ZeraEGF by leaf discs infection as described [31]. For transient transformation, *Nicotiana benthamiana *plants were agroinfiltrated by syringe method into the abaxial side of 3 to 5-week-old leaves [32]. The binary plant vectors pCZeraECFP, pCSPgECFP and pCSPgECFPKDEL were used together with the HC-Pro silencing supressor construct [33].

#### Insect cells

*Spodoptera frugiperda *(Sf9) insect cells (Invitrogen) were grown in suspension or as monolayers at 28°C in serum-free SF900 SFM Medium (Gibco). Recombinant baculovirus were produced by Sf9 co-transfection with flashBAC DNA (from OET) and transfer vectors pBacPak8 containing the Zera-DsRED, DsRED, Zera-EGF or EGF coding sequences according the manufacture's recommendations. Recombinant viruses were titrated [34] and monolayer Sf9 cultures were infected with the recombinant baculovirus at multiplicity of infection (MOI) of 10 to 12.

#### Fungal culture

Expression vectors were introduced into the *T. reesei *strain RutC-30 [35] essentially as described [36] and transformants were selected on plates containing 125 μg/ml of hygromycin B. The transformants were streaked on selective medium containing lactose for induced expression and screened by fluorescence microscopy. Mycelia from the transformants producing the highest amounts of GFP were harvested by filtration.

### Protein extraction and immunoanalysis

Total soluble proteins (TSPs) from transfected cells and tissues were extracted in lysis buffer containing 0.5% SDS and 200 mM DTT for 1 hr at room temperature. The various resulting extracts were centrifuged at 10000 × g for 30 min at 4°C and TSPs were separated on 12 to 15% SDS polyacrylamide gels and the proteins were detected by staining or by immunoblot using the indicated antibodies. The αR8 antiserum was raised in rabbits injected with the synthetic γ zein repeat domain (PPPVHLx8) coupled to the keyhole limpet haemocyanin (KLH) protein used as a carrier. Anti-GFP IgGs were from Molecular Probes. Anti-BiP antiserum was raised in rabbits injected with the recombinant C-terminal half (without the HDEL sequence) of tobacco BiP sequence produced in *E. coli*. Anti-Ct antiserum was raised in rabbits injected with a synthetic calcitonin peptide coupled to the KLH protein. Rabbit polyclonal anti-calnexin, anti-EGF and anti-DsRED were from Abcam. Anti-hGH was raised in rabbits injected with synthetic human growth hormone (hGH, kindly provided by Dr E Giralt, University of Barcelona). To quantify the accumulation level of Zera fusions, proteins were extracted from biomass (frozen leaves and Sf9 cell pellets) with EX buffer (100 mM Tris-HCl pH 8, 50 mM KCl, 5 mM MgCl_2_, 10 mM EDTA, 0.5 M NaCl) in a ratio 1:10 (w/v). An aliquot of the samples was diluted 1:50 in ELISA buffer (12.5 mM sodium borate, 0.1% SDS, 50 mM TCEP, 8 M urea) and quantified by ELISA (NUNC Maxi Sorp plates) using an α-R8 antibody and purified Zera-EGF and Zera-hGH as standards. EGF and hGH expressed without Zera were quantified by ELISA using the respective commercial kits from Peprotech and Roche and following the manufacturers protocol.

### Viability and proliferation analysis

Viability assays were done by using the Vybrant Apoptosis Assay from Molecular Probes and following the manufacturer instructions. Proliferation profiles were determined by using the CyQuant Cell Proliferation Assay (Invitrogen) as indicated by the manufacturers.

### Subcellular fractionation

Transfected mammalian cells were suspended (2 to 3 × 10^6 ^cells/ml) in homogenization buffer HA (10 mM Triethanolamina (TEA) pH 7.4, 1 mM EDTA, 200 μM PMSF) containing 8.5% (w/w) sucrose and were homogenized in a Dounce homogenizer by around 30 up and down strokes. Cell rupture was monitored by staining with 0.005% (w/v) trypan blue and finally the homogenate was clarified by centrifugation for 5 min at 300 × g. Infected Sf9 insect cells (1 to 2 × 10^6 ^cells/ml) were homogenated on PBP5 Buffer (10 mM Hepes pH 7.4, 2 mM EDTA) containing 10% (w/w) sucrose and Complete Protease Inhibitor Cocktail (Invitrogen) using a Potter-Elvehjem homogenizer during 6 min in ice. Transfected *Trichoderma *filamentous fungi were ground in a mortar with liquid nitrogen and homogenized in PBP5 Buffer in a polytron (IKA T25 Basic) for 1 min at 12000 rpm on ice. To remove tissue debris, the suspension was centrifuged 10 min at 200 × g. Transgenic tobacco leaves were ground in a mortar at 0°C in the HB homogenization buffer (Tris 100 mM pH 8, KCl 50 mM, MgCl_2 _5 mM, EDTA 1 mM) containing 10% (w/v) sucrose and protease inhibitors. The homogenate was additionally ground using a polytron (IKA T25 Basic, 10 strokes at 12000 rpm) and filtered through four layers of Miracloth (22 to 24 μm, Calbiochem) to remove tissue debris before to be centrifuged at 50 × g for 5 min at 4°C. The resulting clarified homogenates from the various tissues were loaded onto multistep sucrose gradients (1.08, 1.13, 1.18 and 1.26 g/cm^3^) buffered with the corresponding homogenization buffer. The gradients were centrifuged at 4°C for 2 hr at 80000 × g in a Beckman SW40 Ti rotor. Equivalent aliquots of supernatant, interphase fractions and pellet were analyzed by SDS-PAGE and immunoblot by using specific antibodies. Electrophoretic protein patterns were also analyzed by Coomassie blue or silver staining.

### Immunocytochemistry and imaging

#### Confocal microscopy

Transfected mammalian and insect cells were fixed for 10 min in 3.7% paraformaldehyde and after washing were incubated with specific antibodies for 1 hr. The primary antibodies were detected with anti-rabbit antibodies conjugated to Alexa Fluor 488 or Alexa Fluor 555 dyes (Molecular Probes). Cells expressing fluorescent reporters were simply washed and fixed. Sections of leaf tobacco tissues and *T. reesei *hyphae transformed with fluorescent-derived sequences were mounted in water or Mowiol for direct confocal observation. Micrographs were obtained by using the confocal laser scanning microscope Leica TCS SP (Heidelberg, Germany).

Green fluorescent images were collected at 488 nm excitation using an emission window set at 495 to 535 nm. Red fluorescent images were collected after 543 nm excitation using a 550 to 600 nm emission window. Cyan fluorescent images were collected at 458 nm excitation and an emission window of 470 to 530 nm.

#### Electron microscopy

Mammalian cells from wild-type CHO and p3.1ZerahGH stably transfected cells were fixed at 4°C with 2.5% glutaraldehyde in PHB (100 mM phosphate buffer pH 7.4). After washing, the samples were postfixed at 4°C with 1% osmium tetroxide in PHB containing 0.8% potassium ferricyanide. Then the samples were dehydrated in acetone, infiltrated with Epon resin for 2 days, embedded in the same resin and polymerised at 60°C for 48 hr. Ultrathin sections stained with 2% uranyl acetate and lead citrate were observed in a JEM-1010 electron microscope (Jeol, Japan). For immunolabeling wild-type and transfected CHO cells, ultrathin cryosections were prepared using an ultracryomicrotome (Leica EM FCS, Austria). Ultrathin sections were incubated with antibodies anti-hGH (1/100) or αR8 antiserum (1/700) and protein A-colloidal gold (10 nm). Sections were observed in a JEM-1010 electron microscope (Jeol, Japan). Leaves from wild-type and p19ZeraCt stably transformed tobacco plants were fixed by vacuum infiltration with 1% glutaraldehyde and 2.5% paraformaldehyde in 20 mM phosphate buffer, pH 7.4, for 1 hr at room temperature. Samples were dehydrated and embedded in Lowicryl K4M resin. Immunochemistry was performed essentially as described in [12] using antibodies anti-Ct (1/500), anti-BiP (1/500), and anti-γ zein (1/1500) and protein A-colloidal gold (15 nm). Sections were examined under an electron microscope (Phillips EM301, Eindhoven, The Netherlans). In all cases non-immune serum was used as control.

## Authors' contributions

MT and BL participated in experimental design and performed transfections and protein body isolation from mammalian and Sf9 cells, respectively. SLR performed calcitonin production in tobacco plants. ILT performed transient transformations in plants and confocal microscopy. MB and PM participated in protein body isolation and protein purification experiments. AW and MS designed and performed *Trichoderma reseii *transformations. MDL performed immunoelectron microscopy. MDL and PBH designed the approach, supervised the work and wrote the manuscript. All authors read and approved the final manuscript.
